# Electron and proton magnetic resonance spectroscopic investigation of anthracene oxidation

**DOI:** 10.1016/j.heliyon.2021.e08474

**Published:** 2021-11-25

**Authors:** Mohamed A. Morsy, Abdel-Nasser M. Kawde, Muhammad Kamran, Thomas F. Garrison, Wissam Iali, Salman S. Alharthi

**Affiliations:** a Chemistry Department, College of Chemicals and Materials, King Fahd University of Petroleum & Minerals, P.O. Box 1624, Dhahran 31261, Saudi Arabia; b Department of Chemistry, College of Sciences, Research Institute of Sciences and Engineering, University of Sharjah, P.O. Box 27272, Sharjah, United Arab Emirates; c Department of Chemistry, College of Science, Taif University, P.O. Box 11099, Taif 21944, Saudi Arabia

**Keywords:** EPR Spectroscopy, NMR Spectroscopy, Anthracene cation radical, Radical kinetics

## Abstract

The work reports a method for monitoring anthracene radical-mediated oxidation reactions using electron paramagnetic resonance (EPR) spectroscopy. The formation of anthracene dimer product was well-defined using ^1^H-NMR and ^1^H–^1^H correlation spectroscopy (COSY). Unrestricted 3-21G/B3LYP DFT was used to estimate radical hyperfine spacing (hfs), then to identify the characteristic EPR-spin transitions of anthracene radical intermediate. A detailed investigation of an anthracene oxidation reaction and its possible reaction mechanism in concentrated sulphuric acid is conducted as a model system for polyaromatic hydrocarbons. Peak-to-peak (p2p) intensities of selected EPR-spectral lines were used to evaluate anthracene's oxidation kinetic model. The findings showed that radical intermediate formation is a unimolecular autocatalytic process, dimerization is a pseudo-zero-order reaction, and the latter is the rate-determining step with a half-life of 48 ± 2 min at 25.0 °C.

## Graphical abstract

**Figure:**
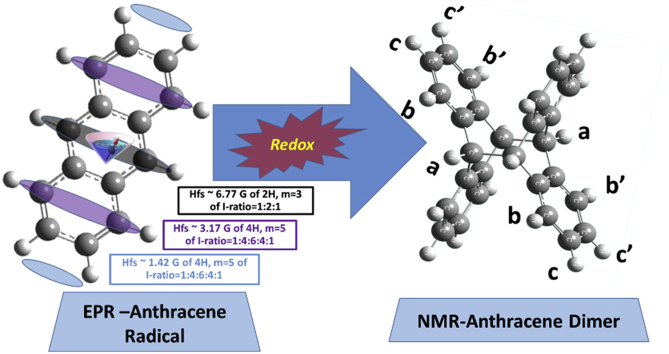

## 1 Introduction

Polycyclic aromatic hydrocarbons (PAHs) are chemicals commonly found in fossil fuels such as oil and coal that are released into the environment through incomplete combustion [1, 2]. Detection of anthracenes, phenanthrenes, benzo-phenanthrenes, fluorenes, chrysenes, pyrenes, perlenes, and coronene in virgin petroleum have been reported in several studies [3, 4, 5]. Many PAHs, such as benzo(a)pyrene, benzo(c)phenanthrene, and 3-methylchloroantrene, are known to be hazardous and potential carcinogens [6, 7]. Furthermore, anthracene has many chemical applications, including its use as a dye and in devices such as optoelectronic and chemosensor [8, 9, 10].

Anthracene has been proffered by Wei et al. [11] as a model compound to investigate the reaction mechanisms of oxidation of condensed aromatics in coals and liquid fuels. One of the limitations of their proposed mechanism was that it focused only on the final products. In their mechanism, there is a possibility that chlorination of anthracene was occurring as a side reaction since the experiments were conducted in an aqueous sodium hypochloride solution [11]. Moreover, while extensive research studies were carried out on the irradiation of anthracene [12, 13, 14, 15], chemical oxidation received little attention [16, 17, 18]. To date, the exact mechanism of anthracene oxidation in acid has not been fully explained.

Due to the growing interest in radical detection and identification, electron paramagnetic resonance (EPR) spectroscopy is uniquely suited as a technique for monitoring free radical kinetics. We have previously reported using EPR spectroscopy as an analytical tool for free radical intermediates generated by different oxidation processes such as the irradiation of alanine [19] and the chemical and electrochemical oxidations of active ingredients in drug formulations [20, 21]. In this work, we report the investigation of the free radical reactions of anthracene in concentrated sulfuric acid using EPR spectroscopy, which can be seen as a model system for other PAHs. Our study delves into a detailed discussion on the anthracene radical's ring proton coupling constants, their density functional theory (DFT) simulation, and their employment in the EPR simulation software to simulate the EPR spectrum [22]. Moreover, the EPR results were analyzed to propose a radical reaction mechanism and estimate the apparent rate constants within the range of 15–40 °C, the half-life periods, and thermodynamic parameters of the activated complexes at 25 °C. The anthracene dimer's formation was confirmed through in situ nuclear magnetic resonance (NMR) spectroscopy experiments.

## 2 Materials and methods

### 2.1 Materials

Synthesis grade anthracene (>96% content), obtained from Merck Schuchardt OHG (Hohenbrunn, DEU), was used after proper mortar grinding into fine, homogenous solid particles. Pure sulfuric acid (H_2_SO_4_, assay 97%), purchased from VWR International (Radnor, PA, USA), was used for anthracene oxidation from its solid state at room temperature.

### 2.2 Sample preparation

For the reaction mixture preparation, 2 mg anthracene was transferred to a 2 mL self-standing polyethylene micro-tube with a screw cap, followed by adding 1 mL of sulfuric acid using a disposable polyethylene graduated pipette. The mixture was thoroughly mixed by shaking for a few seconds. Then approximately 20 μL was transferred into a 50.0 μL disposable glass micropipette (Brand GMBH, Wertheim, DEU) via capillary action for EPR Testing. The micropipette was sealed with a hematocrit sealing compound (Brand GMBH, Wertheim, DEU). For NMR experiments, the remaining portion of the reaction mixture was transferred directly into a regular 5-mm NMR tube.

### 2.3 EPR and ^1^H-NMR spectroscopy

The sealed capillary tube was positioned into a pre-tuned EPR-resonator equipped with a 120 mm (length) x 3 mm (OD) EPR-tube for reproducible runs. An EPR benchtop SPINSCAN-X spectrometer (ADANI, Minsk, BLR) operating at the CW X-band, modulation amplitude = 0.010 mT (0.10 G), and microwave power = 100 mW was employed in this work. The ability of the EPR spectrophotometer to auto-tune and auto-gain allowed for a better signal-to-noise ratio, lower detection limit, and a linear correlation between spin quantity and p2p intensity. The temperature dependence of the reaction in the range between 15 to 40 °C was investigated using a temperature control setup capable of maintaining the temperature of the EPR-sample within ±0.02 °C. Detailed information about the temperature controller, sample holder, and procedure adopted to minimize the temperature gradient across the sample has been described previously [23]. On the other hand, anthracene chloroform solution or the collected portion of the reaction mixture transferred into a regular 5-mm NMR tube was used directly for the NMR experiment. ^1^H-NMR spectroscopy was performed for in situ monitoring of dimer formation and characterization of the product and the monomer anthracene using a Bruker NMR 400 MHz Avance III spectrometer (Bruker Corporation, Billerica, MA, USA).

### 2.4 Computational method

With the ready availability of fast processors for personal computers, DFT calculations on molecules with 15 or more atoms may be completed in a timespan of minutes. Gaussian View 5.0 software was used to construct an approximate geometry for the anthracene molecular system [24]. Geometry optimization calculations were initially carried out at the LCAO-MO-SCF unrestricted Density Function Theory (DFT) level using Becke-Lee-Yang-Parr (BLYP) with the minimal Pople valance-shell STO-3G basis set of Gaussian 09 [25]. The final optimization was carried out by applying Becke's nonlocal three-parameter exchange with Lee-Yang-Parr correlation (B3LYP) functional method using the most common basis set (3-21G). The final optimized molecular model of positively charged anthracene was then employed for the EPR-II basis sets of Barobe. The EPR hyperfine coupling constants were computed with the DFT-B3LYP method by using additional keywords (prop = epr) [25]. GaussView was used to visualize the spin density of the alpha and beta molecular oribals of the unpaired electron at the molecular orbital 47. These computed coupling constants were then imported into the WinSim program from the National Institute of Environmental Health Sciences (NIEHS) [22] to simulate the anthracene radical EPR spectrum.

## 3 Results and discussion

### 3.1 Anthracene radical detection and characterization

**Figure 1:**
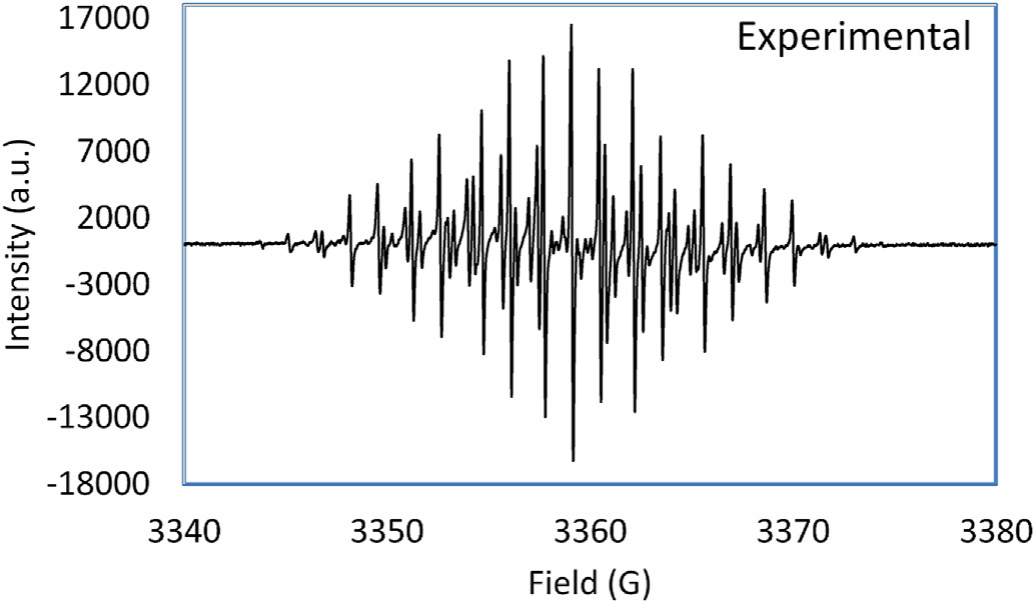
The observed EPR spectrum of anthracene cation radical in concentrated sulfuric acid at room temperature.

Figure 1 shows the EPR spectrum of the reaction mixture obtained using sweep width (SW) = ± 2 mT (±20 G) around the center field, modulation amplitude = 0.010 mT (0.10 G), microwave power = 100 mW, and sweep time = 300 s, during 5 min of the mixing time.

**Figure 2:**
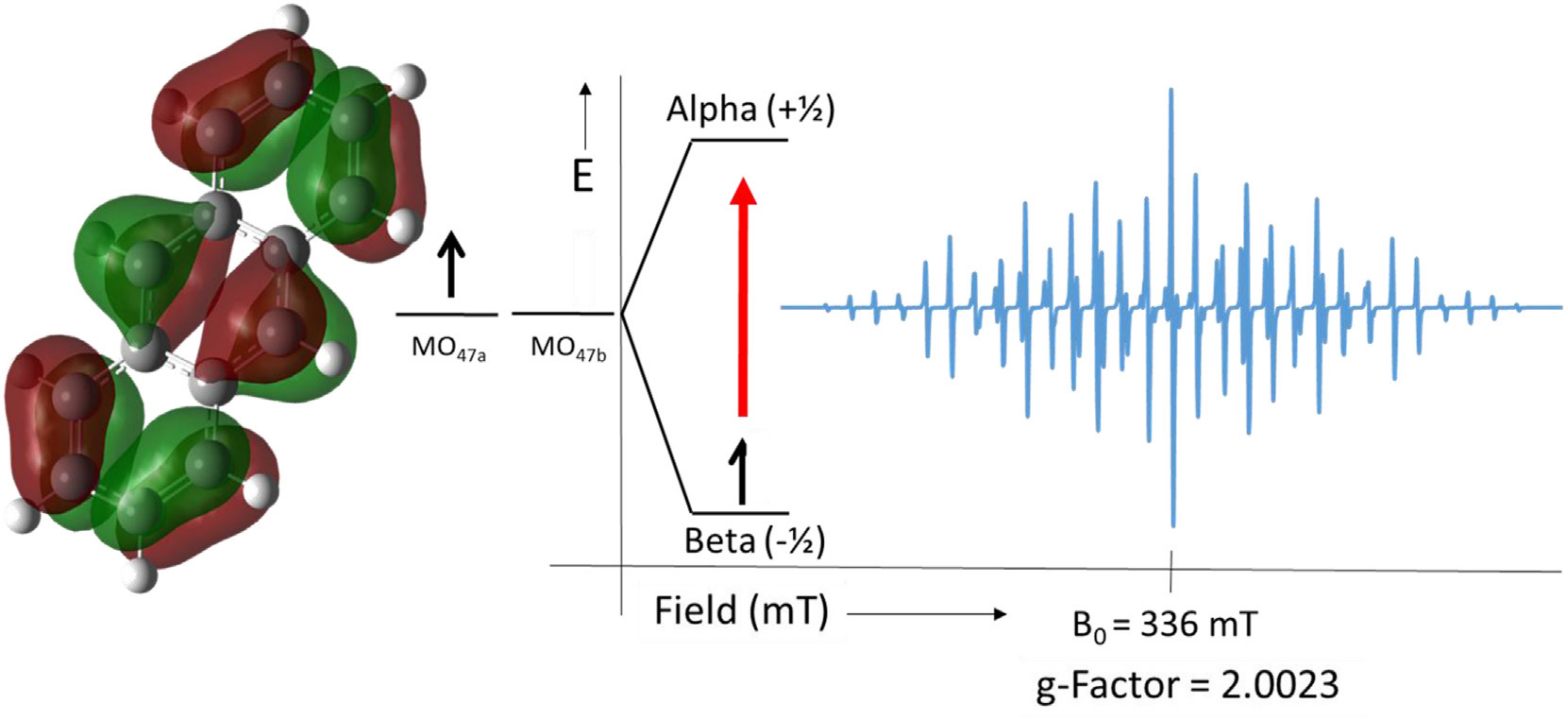
Predicted MO_47_ surface of anthracene radical using GaussView 5.0 of 3–21G UB3LYP DFT calculation and its simulated EPR-spectrum using the estimated hfs in Table 1.

The well-defined hyperfine pattern spectrum shown in Figure 1 is attributed to a simple one-electron oxidized form of anthracene that is consistent with the delocalization of π -electrons in the fused rings of anthracene [24]. Simulated molecular orbital and spin density surfaces of the anthracene radical cation at unrestricted 3-21G/B3LYP DFT level of calculations were obtained using the GaussView 5.0 software [25, 26] and presented in Figure 2. The resulting molecular surface of the cation radical endorse the electron delocalization in both alpha or beta spins includes all ten of the hydrogen nuclei in the anthracene radical, which confirms their contribution to the electron-nucleus spin interaction.

**Table 1:** Summary of unpaired electron-protons total spin, multiplicity, and calculated and experimental hyperfine (hfs) coupling constants of anthracene.

Hydrogen position	Total-spin (S)	Multiplicity 2S + 1 (ratio)	Experimental (hfs)/Gauss	3-21G B3LYP (hfs)/Gauss
9,10	2 x ½ = 1	3 (1:2:1)	6.49	6.34
1,4,5,8	4 x ½ = 2	5 (1:4:6:4:1)	3.04	3.29
2,3,6,7	4 x ½ = 2	5 (1:4:6:4:1)	1.37	1.06

The EPR calculations evinced that the hydrogen atoms are assigned into three sets of equivalent nuclei: (1) two hydrogens at the 9 and 10 carbon positions, (2) four hydrogen atoms at the 1, 4, 5, and 8 carbon positions, and (3) four hydrogen atoms at 2, 3, 6 and 7 carbon positions. Applying this information and the value of ½ for the hydrogen spin with the general isotopic hyperfine line prediction equation (Equation 1) predicted that the observed spectra in Figure 1 should contain 75 lines.

(1) $$\text{No. ​of ​lines ​}=\text{ ​}\overset{N}{\underset{i=1}{\Pi }}\left(2M_{i}I_{i}+1\right)$$

where *M*_i_ is the number of the equivalent nuclei of spin *I*_i_.

**Figure 3:**
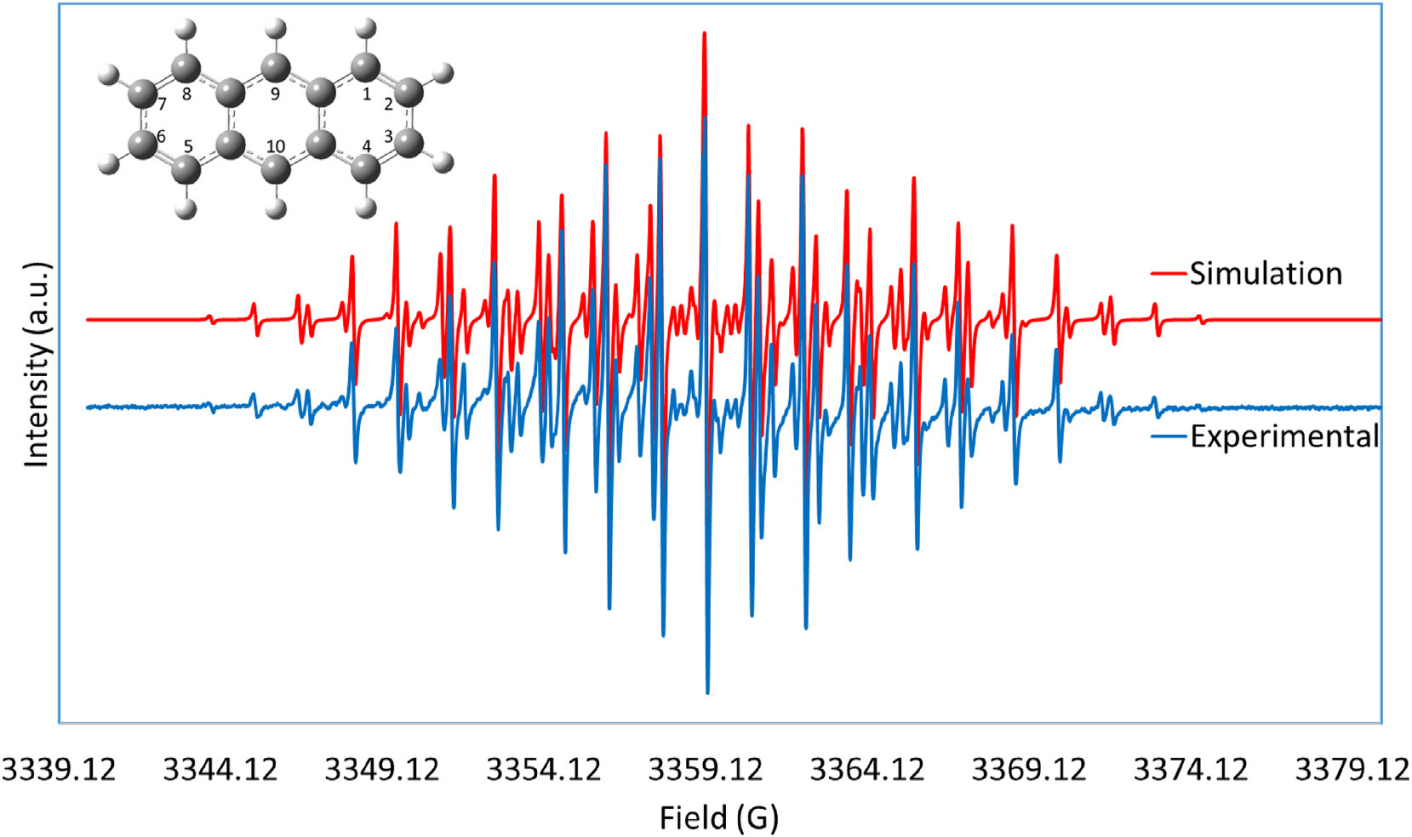
Superimposed experimental and simulated EPR spectra of anthracene radical cation using WinSim software.

Although the spectrum was too complex to assign hyperfine spacing (hfs) for the 75 lines, WinSim-EPR software available from the National Institute of Environmental Health Sciences (NIEHS) [22], simulated the anthracene EPR spectrum. Previous studies have substantiated the ability of the WinSim-EPR software to simulate the EPR spectrum of other complex radical systems [20, 21]. The superimposed spectra in Figure 3 display the close fit between the experimental and simulated EPR spectra.

The simulated results reproduced the precise positions and intensities of the 75-EPR-lines in close agreement with the experimentally obtained spectrum. Moreover, the hfs-values from the simulation program based on the experimental fitting produced that exact hfs values, as reported in Table 1. These results confirm that a simple one-electron anthracene cation free radical system was formed during the dimerization process. This finding agrees with recently published studies on aromatic radical cations in Friedel–Crafts alkylation reactions [27, 28].

### 3.2 Anthracene dimer detection and characterization

**Figure 4:**
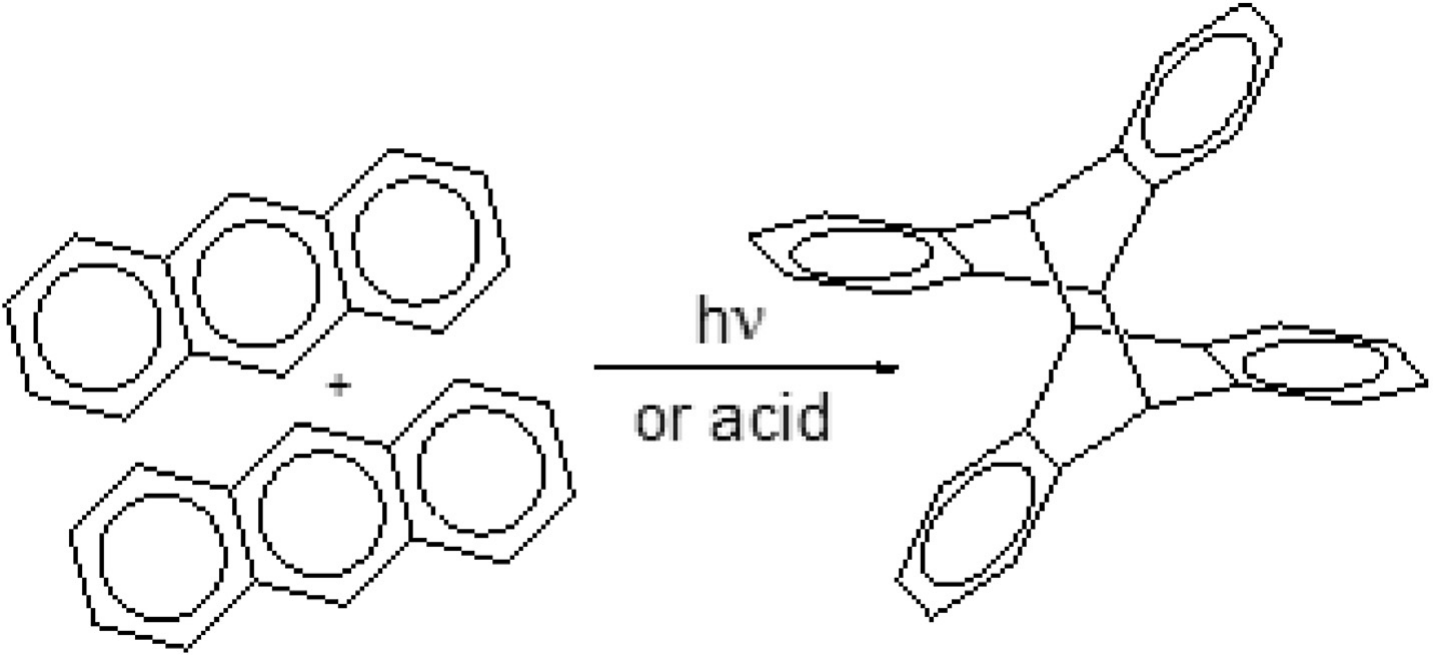
A 4 + 4 cycloaddition of anthracene dimerization reaction.

Photo dimerization and electrophilic substitution at the 9th and 10th carbon positions are two well-known reactions of anthracene. Luther and Weigert reported photo dimerization of anthracene in 1905 [29]. X-ray experiments conducted on solid anthracene by O'Donnell in 1968 found that only dipara-anthracene was formed, and no other intermediates were identified [13]. Dimer formation occurs via an anthracene radical intermediate, through either chemical oxidation [16] or UV-irradiation [14], which undergoes a 4 + 4 cycloaddition, as shown in Figure 4.

In the 1950s, Hirschon et al. attributed anthracene dimerization to the formation of a biradical of the protonated bi-anthracene [16]. Around the same time, Yokosawa and Miyashita related the paramagnetic properties of the oxidized aromatic hydrocarbons to the formation of a triplet state [17]. Other researchers have established that acid oxidation of anthracene produces a simple mono-positive ion [18].

**Figure 5:**
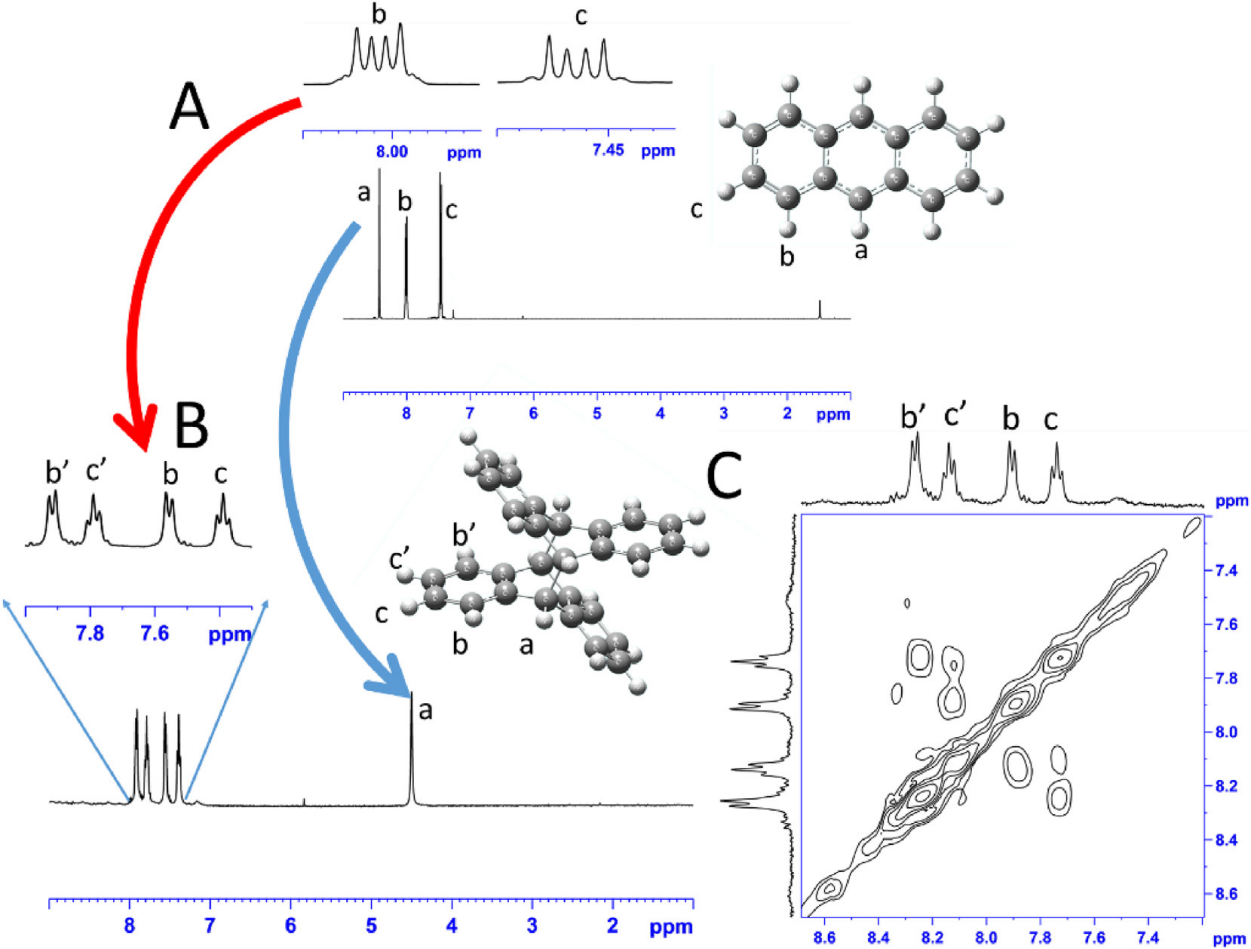
Room temperature 1H NMR spectrum (A) of anthracene starting material dissolved in deuterated chloroform (top) and the in situ dimerization reaction mixture of anthracene in concentrated sulfuric acid (Bottom) and its 1H–1H COSY experiment (B).

In this work, in situ ^1^H NMR and ^1^H–^1^H correlation spectroscopy (COSY) experiments were employed to monitor the reaction progress and confirm that dimer formation occurs in the anthracene/sulfuric acid reaction mixture (see Figure 5).

Figure 5-Part A is of anthracene in chloroform, and Part B and C are of the anthracene-sulphuric acid reaction mixture. The neat 1H NMR spectra of the reaction mixture revealed the formation of anthracene dimer by the complete disappearance of aromatic singlet protons (labeled “a”, s, 2Hs) at δ 9.2 of pure anthracene chloroform solution in Figure 5A and the appearance of the aliphatic protons (also labeled “a”, s, 4Hs) at δ 4.5 of dimer in Figure 5B (blue arrow). This was accompanied by the changing of the aromatic protons “b and c” from multiplet peaks at δ 7.55 and 8.05 into the characteristic doublet-of-doublet peaks at δ 7.53 and 7.83, respectively, of types “c, b, c’, and b’” (red arrow).

Since we aim to see the reaction between the sulfuric acid and anthracene in real-time, we did not use any solvent, and as a consequence, the lock was turned off during the acquisition periods. The dimer anthracene is then obtained in an ionic solution (pure H_2_SO_4_), which is not an inert solvent. The ionic solvent effect was used very well in the literature to characterize materials and for dynamic behavior [30]. The chemical shift is calibrated based on the highest peak, i.e., the sulphuric acid solvent peak. In such an ionic solution (which is the used solvent), a possibility of an ion association (formation of ion-pair) will, of course, affect the NMR spectra (peaks and chemical shift). The obtained 1H NMR spectrum for the dimer anthracene in pure sulfuric acid is characterized by four peaks of the doublet-of-doublet in aromatic region at δ 7.85 (d, 4H, 3J = 7.5 Hz) 7.80 (t, 4H, 3J = 7.5 Hz), 7.55 (d, 4H, 3J = 7.5 Hz) and 7.50 (t, 4H, 3J = 7.5 Hz). The 3J coupling constant for aromatic protons is between 7 and 8 Hz. The observed spectra are comparable to the reported photocatalyzed anthracene products [31]. However, the formation of ionic pair ion association affected the chemical shift of the protons. In addition, the formation of ion-pair will break the symmetry of the molecule, and as a consequence, the NMR overlapped peak of the product will split out. Finally, the 1H–1H COSY spectrum, shown in Figure 5C, confirms that anthracene dimer is the only product formed in the reaction mixture.

### 3.3 Anthracene dimerization reaction kinetics

The ^1^H NMR, ^1^H–^1^H COSY, and EPR results along with the detected bubbles on the solid anthracene particles’ surface support the following overall reaction of anthracene (C_10_H_10_) into an anthracene dimer (C_20_H_20_):

2 C_10_H_10_(s) + H_2_SO_4_(conc) → C_20_H_20_ + H_2_O(l) + SO_2_(g) + ½ O_2_(g)

which can be used to propose the following reaction mechanism for anthracene oxidation with concentrated sulphuric acid:

1 Sulfuric acid solution autoprotolysis (K_1_ (25 ^ο^C) = 2.7 × 10^−4^) [32]:

$$2{\text{H}}_{2}{\text{SO}}_{4}\left(\text{conc}\right)⇌{\text{H}}_{3}{\text{SO}}_{4}^{+}+{\text{HSO}}_{4}^{-}$$

2 Acid diffusion and radical formation (*k*_2_ >> *k*_3_):

$${\text{C}}_{10}{\text{H}}_{10}\left(\text{s}\right)+{\text{H}}_{3}{\text{SO}}_{4}^{+}\overset{k_{2}}{\to }{\text{C}}_{10}{\text{H}}_{10}-{\text{H}}_{3}{\text{SO}}_{4}^{+}$$

C10H10-H3SO4+→k3C10H11⋅++H2O(1)+SO2(g)+12O2(g)

3 Dimer formation (termination/slowest rate processes):

$${\text{C}}_{10}{\text{H}}_{10}\left(\text{s}\right){+\text{C}}_{10}{\text{H}}_{11}\cdot^{+}{\text{HSO}}_{4}^{-}\overset{k_{4}}{\to }{\text{C}}_{20}{\text{H}}_{20}+{\text{H}}_{2}{\text{SO}}_{4}\left(\text{conc}\right)$$

The salient feature of the proposed mechanism is the dissolution of anthracene in the concentrated sulfuric acid in the form of anthracene radical cations via a consecutive step starting with a diffusion process (*k*_2_) then a redox reaction (*k*_3_), where *k*_2_ >> *k*_3_, as the radical cation was the only species detected. The radical formation step is then followed by the dimer formation step (*k*_4_, the slowest step) toward the final products of the anthracene dimer.

**Figure 6:**
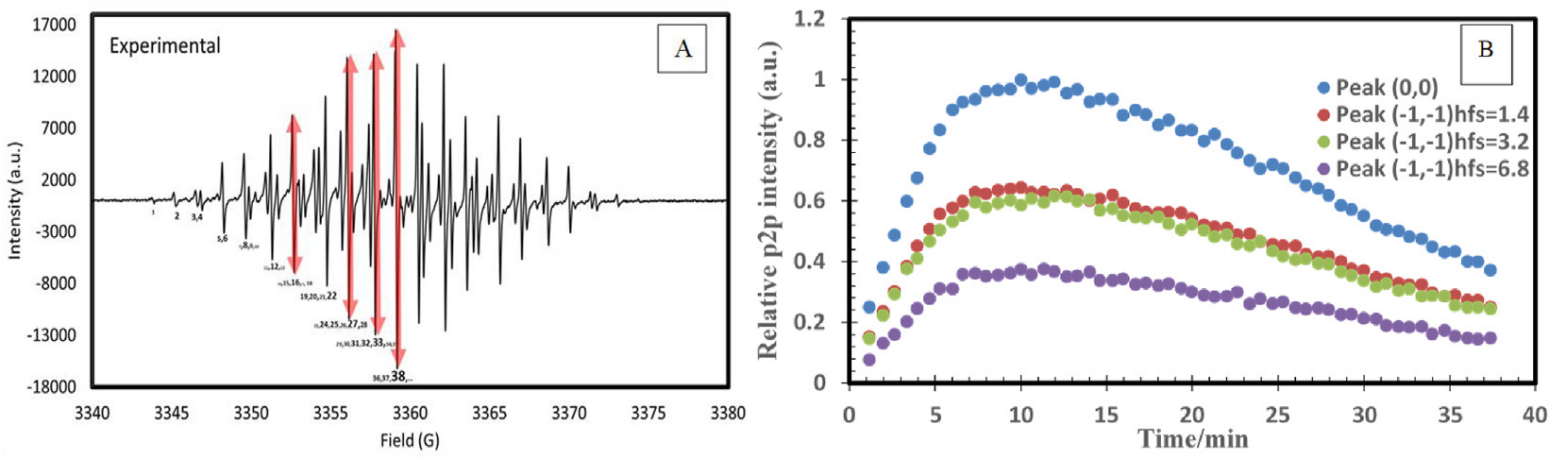
Four selected peaks of different spin-coupling transitions (highlighted in red, part (A) produce similar relative p2p-time trends from 54 spectral runs of anthracene radical (B), confirming their suitability for radical kinetic investigation.

Specific peak-to-peak (p2p) intensities from the anthracene EPR spectrum may be selected to relatively quantify anthracene's radical concentrations, which can subsequently be utilized to analyze the reaction kinetics. Four intense peaks numbered 16, 27, 33 and 38 were screened, which correspond to the (−1,-1) hfs = 6.8, (−1,-1) hfs = 3.2, (−1,-1) hfs = 1.4, and (0,0) spin-spin transitions, respectively (highlighted in red in Figure 6A, from left to right, respectively). Their relative peak intensities to the normalized intensity of (0,0) peak at ∼ 10 min are presented in Figure 6B over 40 min at 40 s time intervals. The results indicated that any of the selected peaks are associated with the radical intermediate processes, namely radical formation and radical decay (also referred to as dimer formation, the only observed product). However, peak-38 (0,0) transition (blue curve in Figure 6B) emerged as the optimal peak for interpreting the reaction kinetics. The analysis of these results also indicated that radical formation dominated during the first 4 min, whereas dimer formation prevailed after 15 min. During the 4–15 min time interval, the peak-38 intensities were exceedingly complex and difficult to resolve. The experimental work extended to cover several experimental trials conducted at different ratios of anthracene to sulfuric acid (see Table 2). Data points were also collected at time intervals ranging from 20 to 40 s, depending on the temperature of the experiment.

**Table 2:** Different EPR-peaks having different hfs, interval time (IT), and the used kinetic models are used to estimate the rate constant and half-life period of the radical intermediate formation and its decay toward dimer formation.

Anthracene/Acid/IT Peaks No.	Radical Formation “Intermediate” $\ln\left(\frac{\alpha }{1-\alpha }\right)=\pm \;k_{3}\left(t-t_{\frac{1}{2}}\right)$	Radical Decay “Dimer Formation” (α/α_0_ – 1) = − (*k*_zo_/α_0_) = − *k*_4_*t*
2 mg/1 mL/40 s		
Peak-38:	Y = 0.69 X – 2.07	Y = − 0.0191 X + 1
Peak-33:	Y = 0.72 X – 1.99	Y = − 0.0186 X + 1
Peak-27:	Y = 0.63 X – 1.81	Y = − 0.0192 X + 1
Peak-16:	Y = 0.68 X – 2.07	Y = − 0.0187 X + 1
	Rate Constant Average:	Rate Constant Average:
	*k*_3_ = 0.67 ± 0.04 min^−1^	*k*_4_ = 0.0189 ± 0.001 min^−1^
	t_½_ = 2.9 ± 0.1 min	t_½_ = 26.5 ± 0.5 min
2 mg/1 mL/30 s		
Peak-38:	Y = 0.69 X – 1.65	Y = − 0.0174 X + 1
Peak-33:	Y = 0.64 X – 1.91	Y = − 0.0177 X + 1
Peak-27:	Y = 0.73 X – 1.70	Y = − 0.0163 X + 1
Peak-16:	Y = 0.63 X – 1.85	Y = − 0.0214 X + 1
	Rate Constant Average:	Rate Constant Average:
	*k*_3_ = 0.67 ± 0.05 min^−1^	*k*_4_ = 0.0182 ± 0.001 min^−1^
	*t*_½_ = 2.7 ± 0.4 min	*t*_½_ = 27.5 ± 3.1 min
3 mg/1 mL/40 s		
Peak-38:	Y = 0.99 X – 1.18	Y = − 0.0188 X + 1
Peak-33:	Y = 0.78 X – 1.04	Y = − 0.0197 X + 1
Peak-27:	Y = 0.90 X – 1.55	Y = − 0.0205 X + 1
Peak-16:	Y = 0.97 X – 1.89	Y = − 0.0210 X + 1
	Rate Constant Average:	Rate Constant Average:
	*k*_3_ = 0.91 ± 0.05 min^−1^	*k*_4_ = 0.025 ± 0.001 min^−1^
	*t*_½_ = 1.6 ± 0.4 min	*t*_½_ = 26.0 ± 3.1 min

Widely known, radicals are typically intermediate species with very short lifetimes [32]. In the current study, we have demonstrated that the high polarity and viscosity of concentrated sulfuric acid enable the detection of anthracene radicals and the measurement of their formation and decay rates using EPR spectroscopy at and around room temperatures. The anthracene reaction occurred at the surface of its powder just after its mixing with the acid. Simultaneously, bubbles that may be attributed to SO_2_ and O_2_ gases evolved on the anthracene solid-state surface, indicating a heterogeneous catalytic process. Therefore, many previously reported heterogeneous kinetic models were evaluated [33]. The following two heterogeneous kinetic models (Eqs. (2) and (3)) produced the best fit:

1 Autocatalysis heterogeneous reaction:

(2) ln(α1−α)=±kac(t−t12)

**Figure 7:**
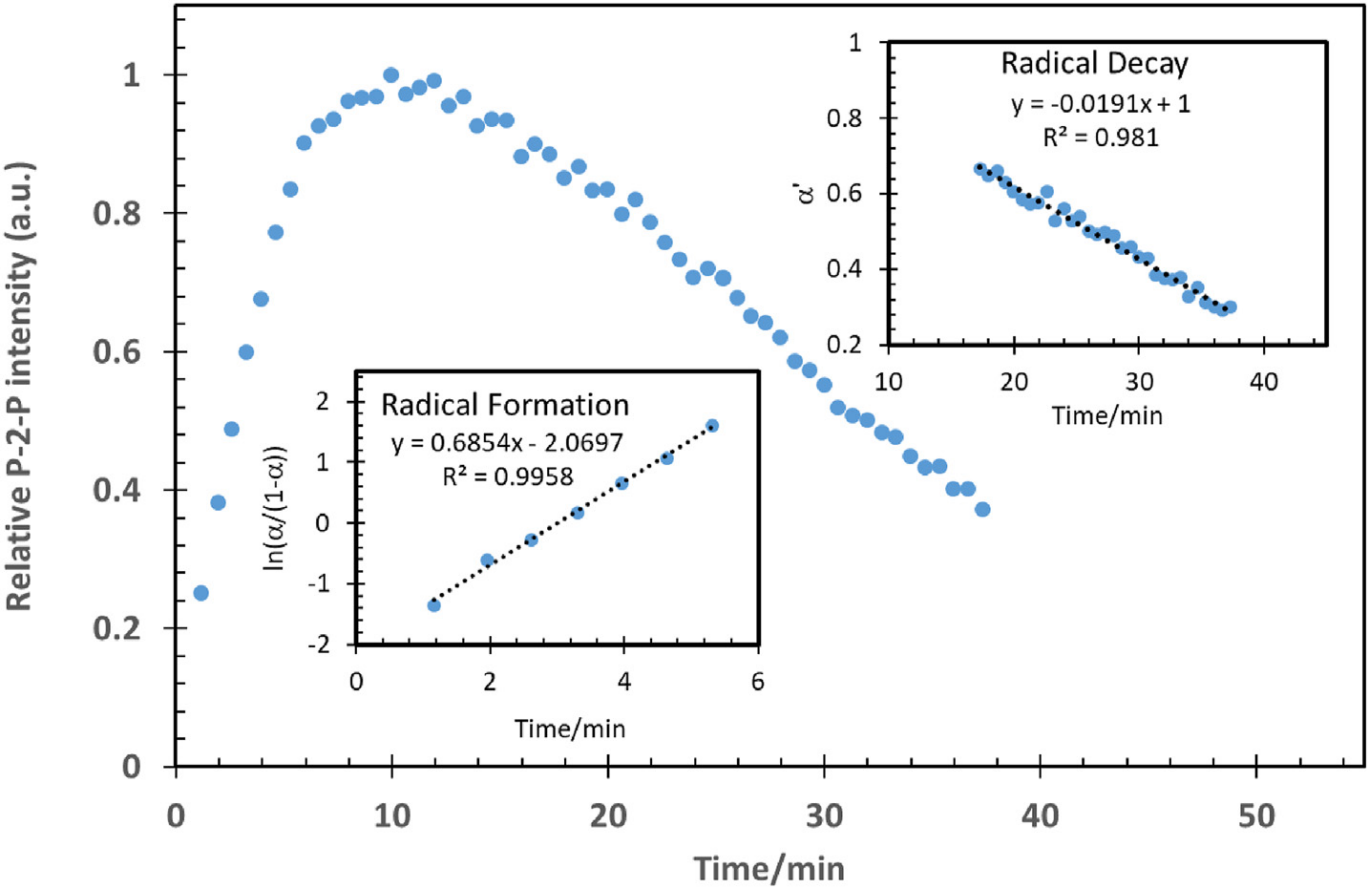
Anthracene radical formation and decay curve analysis using kinetic models (1) and (2) of the radical formation and decay, namely 1st order autocatalysis and pseudo-zero-order, respectively.

2 Zero-order heterogeneous reaction:

(3) $$\left(\alpha -\alpha_{0}\right)=\pm \;k_{zo}\text{ ​t}$$

where α is defined as the fraction of the reacting anthracene species at time *t*, and *k* is the apparent rate constant. For the radical formation, the EPR-signal amplitude (p2p-intensity) is proportional to the formed anthracene radical cation. In contrast, for the radical disappearance, the plot of the EPR-signal intensity (p2p) versus the time, which is apparently a zero-order process, is used to identify the EPR signal amplitude at t = 0. The latter value is proportional to the initial radical intermediate concentration and is equivalent to α_0_ value. The use of the α and α_0_ values versus time in Figure 7 indicate that radical formation (*k*_3_) follows Eq. (2), the autocatalysis heterogeneous model, with a rate constant *k*_3_ = *k*_ac_ = 0.67 min^−1^ and a half-life (*t*_½_) 3 min and the dimer formation appears (*k*_4_) follows Eq. (3), zero-order kinetics, while its proposed kinetics is pseudo-second-order. The large quantity of solid anthracene and the sulphuric acid compared to the generated anthracene radical cation could explain its observed kinetics, which has been described as a pseudo-zero-order heterogeneous model that has a relative rate constant *k*_4_ = (*k*_zo_/α_0_) = 0.019 min^−1^ and a half-life (*t*_½_) approximately 27 min by using 2 mg anthracene in 1 mL concentrated sulfuric acid. These two models were then tested on several samples at the same temperature but with different interval times (IT). Table 2 summarizes the results of the proposed kinetic models (1) and (2) applied to the four essential transitions of peaks 16, 27, 33, and 38. All the peaks produced similar apparent rate constants regardless of interval times (IT) variation.

The results from higher anthracene concentration relative to the sulphuric acid indicated that increases of anthracene reduced the half –life period of the radical formation. At the same time, the rate of the radical decay (*i.e.* dimerization of anthracene) is almost unaffected, as shown in Table 2. After evaluating the different reaction compositions, the ratio of 2 mg/1 mL was selected for studying the effect of temperature based on the half-life period. Since the oxidation process generated radicals localized on solid surfaces, our selection of kinetic models focused on heterogeneous catalytic processes, especially those related to zero and first order.

**Figure 8:**
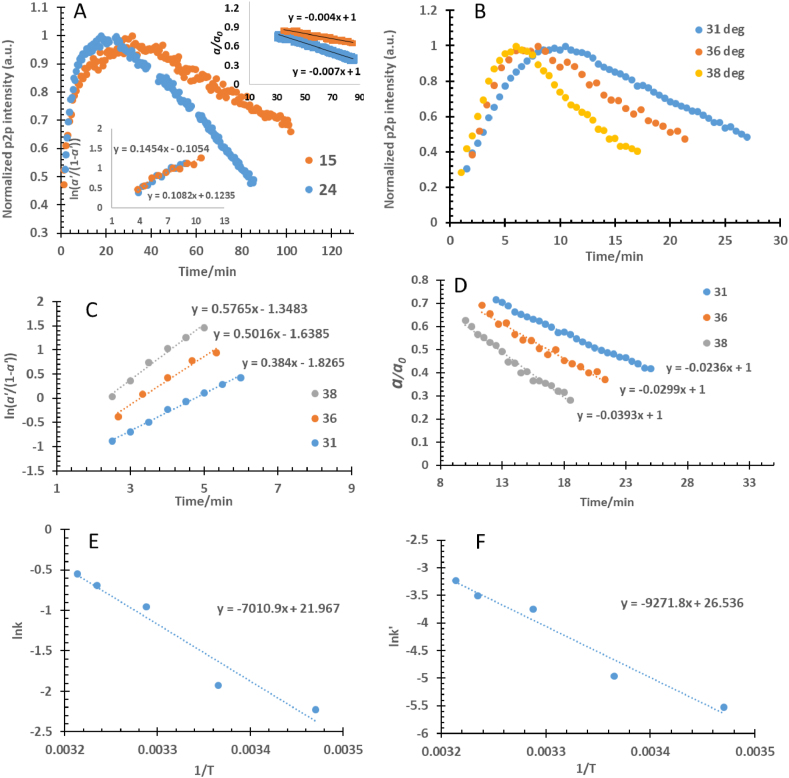
Anthracene radical formation and decay kinetic models at 15 and 24 °C are shown in (A), while the models at 31, 36, and 38 °C are presented in (B), (C), and (D). The graphs (E) and (F) are the Arrhenius plots of the radical intermediate formation and its decay reaction toward dimer formation, respectively.

Figures 8A and B show the p2p-intensity of the obtained EPR spectra of the anthracene radical reactions at different temperatures in the range of 15–40 °C Figures 8A, C, and D show the best linear fit of the autocatalysis heterogeneous unimolecular model to the radical formation and the pseudo-zero-order model to the dimer formation. Figures 8E and F display the Arrhenius plots used to estimate apparent rate constants of the proposed radical processes and the thermodynamic parameters at 25.0 °C of their activated complexes. The observed straight line indicated that both processes, i.e. radical and dimer formations, are single rate-limited thermally activated processes.

Table 3 summarizes the rate constants values and the thermodynamic parameters of the anthracene dimer formation extracted from Figure 8. The results demonstrate that the anthracene dimer formation in sulfuric acid is the rate-determining step having *k*_4_ = (k_zo_/α_0_) = 0.0104 ± 0.0004 min-1 and its formation has a half-life period of about 47.9 ± 1.9 min at 25.0 °C.

**Table 3:** The dependence of the rate constants for radical intermediate formation and decay (dimer formation) processes on the temperature range of 15–40 °C of 2 mg/1 mL Anthracene/Acid mixture and 30 s IT of peak-38, rate constants, half-life periods, and the expected thermodynamic parameters of the activated complex of radical and dimer formation at 25 °C.

Reaction Mixture Temperature in ^o^C	Rate Constant of Radical Formation (*k*_3_/min^−1^)	Rate Constant of Dimer Formation (*k*_4_/min^−1^)
15.0 ± 0.1	0.108 ± 0.010	0.00400 ± 0.0002
24.0 ± 0.1	0.145 ± 0.019	0.00700 ± 0.0005
31.0 ± 0.1	0.384 ± 0.024	0.0236 ± 0.0018
36.0 ± 0.1	0.502 ± 0.035	0.0299 ± 0.0022
38.0 ± 0.1	0.577 ± 0.040	0.0393 ± 0.0026
Rate Constant (k) @ 25 ^ο^C/min^−1^ =	0.213 ± 0.015	0.0104 ± 0.0004
Half-life period (t½ =) @ 25 ^ο^C/min =	3.23 ± 0.22	47.9 ± 1.9
Enthalpy (ΔHǂ) @ 25 ^ο^C/kJ mol^−1^ =	55.8 ± 3.3	89.0 ± 1.6
Entropy (ΔSǂ) @ 25 ^ο^C/J mol^−1^ K^−1^ =	- 70.6 ± 0.7	- 32.6 ± 5.6
Gibbs' Energy (ΔGǂ) @ 25 ^ο^C/kJ mol^−1^ =	76.9 ± 0.02	84.3 ± 0.10

The magnitudes of enthalpy and entropy of activation reflect the transition-state complexes. The negative values of the entropy is likely due to a vibrational degree of freedom loss. This loss is much more in the radical formation process than the radical decay due to reducing the degree of freedom by the bond formation in the dimer. Furthermore, dimer formation was found to be the slowest process because the enthalpy of activation and Gibbs’ activation energies have higher values than those of the radical formation.

## 4 Conclusion

The work presents a direct method for investigating the product of the anthracene oxidation reaction and its kinetics using EPR spectroscopy. The unrestricted smallest split-valence basis set-DFT molecular orbital calculations produced acceptable hfs for the initial estimation of the actual isotropic hyperfine coupling values of paramagnetic species. *In situ* ^1^H NMR and ^1^H–^1^H COSY experiments confirmed the formation of anthracene dimers in the anthracene/sulfuric acid reaction mixture as the sole product. Peak-to-peak (p2p) intensities of non-overlapped EPR spectral lines of the radical intermediate were used to determine the apparent rate constants and half-life periods of radical formation and radical decay.

Moreover, using a precise EPR sample temperature controller enabled the determination of the apparent rate constant, half-life period, and thermodynamic activation parameters of the radical processes at a particular temperature [23]. The findings showed that radical decay (i.e., dimer formation process) is the rate-determining step and has a 48 ± 2 min half-life at 25.0 °C. In comparison, radical formation had a much shorter half-life of 3.23 ± 0.22 min at 25.0 °C. These findings were consistent with enthalpies for activation where the enthalpy of activation for dimer formation (89.0 ± 1.6 kJ mol^−1^ at 25.0 °C) was higher than the enthalpy of activation for radical formation (55.8 ± 3.3 kJ mol^−1^ at 25.0 °C).

The analysis of anthracene oxidation presented in this study will support future kinetic investigations of PAH redox reactions that produce stable radical intermediates. Further investigation is needed to expand the scope of this technique to other important PAH compounds, especially the PAHs that commonly occur as environmental pollutants from fuel combustion. The identification of anthracene dimer degradation products and their kinetics will be considered in future studies.

## Declarations

### Author contribution statement

Mohamed A. Morsy: Conceived and designed the experiments; Performed the experiments; Analyzed and interpreted the data; Contributed reagents, materials, analysis tools or data; Wrote the paper.

Abdel-Nasser M. Kawde: Conceived and designed the experiments; Analyzed and interpreted the data.

Muhammad Kamran; Wissam Iali: Performed the experiments; Analyzed and interpreted the data.

Thomas F. Garrison: Analyzed and interpreted the data; Wrote the paper.

Salman S. Alharthi: Analyzed and interpreted the data; Contributed reagents, materials, analysis tools or data; Wrote the paper.

### Funding statement

This work was supported by Deanship of Scientific Research, 10.13039/501100004055King Fahd university of Petroleum and Minerals (IN161046) and Taif University Researchers Supporting Program, 10.13039/501100006261Taif University (TURSP-2020/90).

### Data availability statement

Data will be made available on request.

### Declaration of interests statement

The authors declare no conflict of interest.

### Additional information

No additional information is available for this paper.
